# Supplementary material for: Non-Occupational Exposure to Pesticides: Experimental Approaches and Analytical Techniques (from 2019)
Source: Molecules. 2021 Jun 16;26(12):3688. doi: 10.3390/molecules26123688 (PMC8235395; doi:10.3390/molecules26123688)
Supplement: Supplementary file 1 [file molecules-26-03688-s001.zip › molecules-1218136-supplementary.pdf]

Non-Occupational Exposure to Pesticides: Experimental Approaches and Analytical Techniques (from 2019)

Lucía Vera-Herrera, Daniele Sadutto and Yolanda Picó*

Food and Environmental Safety Research Group, Desertification Research Centre—CIDE (CSIC-UV-GV), University of Valencia (SAMA-UV), Moncada-Naquera Road, Km 4.5, 46113 Moncada, Spain

*Authors to whom correspondence should be addressed.

Supporting information

Number of pages: 21

Number of tables: 2

List of tables

Table S1: Detailed information about the selected analytical methods published between 2019 and 2021 for the analysis of pesticides in environmental matrices and food to analyse human exposure.

Table S2: Detailed information about the analytical methods published between 2019 and 2021 for the analysis of pesticides in biological matrices and between 2018 and 2021 for the Wastewater-Based Epidemiology (WBE) studies to analyse human exposure to pesticide residues

Table S1. Selected analytical methods published between 2019 and 2021 for the analysis of pesticides in environmental matrices and food to analyse human exposure.

Sample		Nº Pesticides or biomarkers	Sample treatment			Separation and detection			Recovery %	LOD (or LOQ*)	Ref.
Matrix	Volume/ Weight		Method	Extraction	Clean-up	Technique	Separation equipment	Mobile phases			
Drinking water (TPW and tap)	50 ml	7	LLE	30 ml DCM	Purified passing sample through a chromatographic column	UPLC-QqQ-MS/MS: in MRM. ESI +	YMC ODS-AQ (100 mm × 2.1 mm, 3 µm)	Gradient mode: 0.1% FA [phase A] and 100% ACN [B] at 300 µL/min	73 - 94%	30 - 70 ng/L	[52]
Drinking water (Groundwater and tap)	500 ml	16	SPE	Oasis HLB (6 cc/ 500 mg) eluted with 4 ml CAN + 4 ml methanol	-	UPLC-MS/MS: in MRM. ESI +	Waters ACQUITY UPLC SS T3 (100 mm × 2.1 mm, 1.8 µm)	Gradient mode: 0.005% FA [phase A] and 100% ACN [B] at 250 µL/min	74 - 123%	0.01 - 0.2 ng/L*	[53]
Groundwater	1 000 ml	56	LLE	20 ml DCM (x 3): with three pH conditions (6.5-8.0, < 2.0 and > 10.0)	-	GC-MS: SIM and SCAN mode. EI	DB-5MS column (30 m × 0.25 mm; 0.25 µm)	Helium at 1000 µL/min using temperature gradient	70 – 133%	2.5 - 247 (ng/L)	[49]
Tap water	500 ml	9	LLE	70 ml DCM	Silica gel column with anhydrous Na_2SO_4 (CNWBOND 10cc/10g)	GC-MS: In MRM mode. EI	HP-5 ms UI (30 m × 0.25 mm ;0.25 µm)	Nitrogen at 1150 µL/min using temperature gradient	76 - 94%	0.0011 - 0.43 ng/L	[54]
Surface water	1000 ml	65	SPE	C18 (6 cc/1000 mg)	5 ml ethyl acetate + 5 x 2 ml DCM	GC-MS/MS: in SRM EI	DB-5MS (30 m × 0.25 mm; 0.25 µm)	-	-	-	[50]
Sediment and soil	-		PLE	Acetone:DCM (1:1, v/v)	Florisil ® (6cm3/ 1000 mg) eluted with 10 ml acetone/hexane (20/80 v/v)						
Surface water	1000 ml	8	LLE	Ethyl acetate + methylene	-	GC-MS: in SIM EI	TG-5MS (30 m × 0.25 mm; 0.25 µm)	Helium at 1000 µL/min using temperature gradient	80 - 94%	1.05 - 2.60 ppb	[55]
Sediment	5 g		QuEChERS	15 ml ACN 1% AA + 6g $MgSO_4$ + 1.5 g NaOAc	d-SPE with 25 mg PSA + 25 mg C18 +7 mg GCB + 150 mg $MgSO_4$						
Surface water	10 ml	224	-	-	-	DAI to LC-MS/MS: in MRM. ESI + and -	Agilent Zorbax C18 (3.0×100 mm;1.8 µm)	Gradient mode: aqueous FA/ammonium formate [phase A] and methanol [B] at 600 µL/min	34-135%	1.0-106 ng/L	[51]
Sediment	10 g	119	PLE	DCM; purified in a Florisil® (6cm3/1000 mg)	Purification: 1º fraction: DCM + 50:50 DCM: ethyl	GC-MS/MS: in SIM. EI	DB-5MS (30 m × 0.25 mm; 0.25 µm)	Helium at 1200 µL/min	75 - 102%	0.6 - 3.4 µg/kg	

					acetate; 2º fraction: 20% DCM in hexane + 50% ethyl acetate in hexane						
Soil	20 g	21	Soxhlet	Acetone/n-hexane (1:1, v/v)	Florisil cartridge (6cc/1000mg) eluted with 5 ml n-hexane:acetone (95:5, v/v)	GC-MS	DB-5MS (30 m × 0.2 mm; 0.25-μm)	Helium at 1000 μL/min	75.9 - 126.1%	-	[41]
Soil	5 g	23	QuEChERS	10 ml ACN 1% AA + 1g NaOAc + 4 g $MgSO_4$	dSPE with 50 mg PSA + 150 mg $MgSO_4$	Polar compounds: LC-MS/MS: Ionization in + and -<br>A-polar compounds: GC-MS/MS	HSS T3-C18 (2.1 x 100 mm; 1.7 μM)<br>Restek ClPesticides (30m x 0.25 mm; 25 μM)	Gradient mode: water 5mM ammonium formate + 0.1% FA [phase A] and MeOH:H20 95:5 5mM ammonium formate and 0.1% FA [B] at 400 μL/min<br>-	70 - 120%	1 - 10 μg/kg	[56]
Soil	1 g	8	SPE	10 mL cartridge column packed with: Na_2SO_4 (0.5 g)	Florisil (1 g, 60–100 mesh), acidic silica gel (1 g) + copper powder (0.5 g) eluted with 15 ml DCM	GC-ECD: in SIM. EI ionization	HP-5MS (30 m× 0.25 mm; 0.2 5 μm)	Helium at 1200 μL/min	80 - 96%	0.001 - 0.025 ng/g	[39]
Sediment	4 g	8	PLE	Acetone:n-hexane (1:1, v/v); Purified two times: 1) in an activated copper column (20% HCl); 2) ENVI-CARB/PSA cartridge (6cc/500 mg)	3 ml hexane (1º purification); 6 ml hexane-ethyl-acetate (7/3, v/v) (2º purification)	GC-MS: in SIM. EI ionization	HT-8 (25 ml×0.22 mm; 0.25 μm)	Helium at 1000 μL/min	89 - 118%	-	[40]
Air particulates	1344 m^3	46	Soxhlet	Hexane:acetone:MeOH (50:40:10 v:v:v)	-	UPLC-MS/MS: in MRM. ESI in + and -<br>GC-MS-QqQ: in MRM. EI -	Acquity BEH C18 (2.1 x 100 mm; 1.7 μm)<br>DB-35MS capillary column (20 m x 0.18 mm; 0.18 μm)	Gradient mode: 0.1% FA [phase A] and in MeOH [B] at 350 μL/min<br>Helium at 1000 μL/min	72 - 128%	0.04 - 0.1 ng/m^3*	[63]
Air particulates	-	7	SLE	10 ml ethyl acetate:acetone (9:1, v/v)	-	LC-MS/MS: in MRM mode. ESI +	RSpak DE-213 (150 mm x 2mm)	Gradient mode: 0.1% (v/v) FA 10mM AA in water [phase A] and 0.1% (v/v) FA 10mM AA in MeOH [B] at 400 μL/min	-	-	[60]

Air particulates	30 m^3	10	Soxhlet	Acetone	Exchanged into hexane; purified in a silica gel column	GC-MS: EI ionization	J&W DB-5ms Ultra Inert (30 m l × 0.25 mm; 0.25 µm)	Helium at 1150 µL/min	60 - 149%	0.1 - 1 ng/m^3	[61]
Air particulates	432 m^3	26	PUF: Soxhlet<br>GFF: SLE	150 ml n-hexane:acetone (v:v, 1:1)<br>25 ml n-hexane:acetone (v:v, 1:1)	Silica gel/alumina chromatographic column eluted with 70 ml DCM	GC-MS: in SIM mode. Negative chemical ionization (NCI)	HP-5 MS column (30 m × 0.25 mm; 0.25 µm)	Helium at 1000 µL/min	65 - 120%	0.1 - 25.0 pg/m3	[62]
$PM_{2.5}$	158.4 m^3	4	QuEChERS	20 ml ACN	dSPE with 0.4 g PSA	LC-MS/MS-QqQ: in MRM. ESI +	XR-ODS C18 column (50 mm × 2.0 mm; 2.2 µm)	Gradient mode: 5 mmol/L ammonium formate [phase A] and ACN [B] at 200 µL/min	78 - 97%	0.0005 - 0.355 ng/m^3	[59]
$PM_{2.6}$ and PM_{10}	1627.2 m^3	34	SLE	500 µL ethyl acetate/ACN (30:70)	-	GC-MS: in SIM. EI	Rtx®-5MS (29.9 m × 0.25 mm; 0.25 µm)	Helium at 1000 µL/min	90 - 144%	0.14 - 0.44 ng/ml	[58]
Dust (indoor)	10 g	26	Soxhlet	DCM; purified in a silica-alumina column	-	GC-MS-QqQ: in SIM. EI	CP-Sil 8CB (50 m x 0.25 mm; 0.25 µm)	Helium at 1000 µL/min	88 - 110%	1.31 - 7.30 pg/g	[43]
Dust (indoor)	20 g	24	Soxhlet	300 ml DCM; purified in a silica-alumina column	-	GC-MS-QqQ: in SIM. EI	CP-Sil 8CB (50 m × 0.25 mm; 0.25 µm)	Helium at 1000 µL/min	88 - 110%	1.31 - 7.30 pg/g	[44]
Dust (road)	2 g	10	SLE	10 ml acetone + 10 ml acetone:hexane (1:1, v/v); purified in a silica gel column	DCM + hexane (purification)	GC-MS	J&W DB-5ms Ultra Inert (30 m × 0.25 mm; 0.25 µm)	Helium at 1150 µL/min	60 - 120%	0.0010 - 0.010 µg/g	[34]
Silicone wristband	-	75	SLE	50 ml ethyl acetate; purified in a C18 silica colun (500mg)	9 ml ACN (purification)	GC-ECD	Quantitation: DB-17MS (30 m x 0.25 mm, 0.25 µm); Confirmation: DB-XLB (30 m x0.25 mm, 25 µm)	Hydrogen at 2600 µL/min	11 - 142% (median 55%)	0.44 - 20.9 pg/µL	[64]
Fish	3 g	8	PLE	Acetone:n-hexane (1:1, v/v)	Twice:<br>1) with a copper column eluted with hexane<br>2) ENVI-CARB/PSA cartridge eluted with6 ml hexane:ethyl-acetate (7:3, v/v)	GC-MS: in SIM. EI	HT-8 (25 m × 0.22 mm; 0.25 µm)	Helium at 1000 µL/min using temperature gradient	89 - 118%	-	[40]

Fish	3 g	18	Soxhlet	150 ml hexane:acetone (3:1, v/v)	Glass column (30 cm x 1 cm) [1 g neutral alumina + 1 g neutral silica + 8 g acidified silica + 4 g Na_2SO_4] eluted with 50 ml DCM and hexane (1:1, v/v)	GC-µECD	Agilent HP-5 (30 m × 320 µm; 0.25 µm)	Nitrogen at 1625.5 µL/min using temperature gradient	61 - 136 %	0.0003 - 0.0054 ng/g	[69]
Cow´s milk	2 g	18	LLE	3 ml n-hexane:DCM (1:1, v:v)	Glass column (30 cm x 1 cm) [1 g 5% deactivated silica + 1 g 5% deactivated Florisil + 1 g Na_2SO_4] eluted with 15 ml n-hexane + 10 ml DCM	GC-µECD	HP-5, 5% (30-m x 0.32-mm; 0.25-mm)	Helium at 2500 µL/min using temperature gradient	70 - 109%	0.003– 0.63 ng/g	[70]
Wax	20 g	1	QuEChERS	10 ml ACN; NaCl + $MgSO_4$ + sodium citrate + sodium hydrogen citrate sesquihydrate	dSPE (150 mg $MgSO_4$ + 25 mg C_{18} + 25 mg PSA)	LC-MS/MS: In MRM. ESI -	Synergi Hydro RP® column	-	95%	20 µg/kg *	[71]
Wax	1 g	More than 600	QuEChERS	10 ml water + 10 ml CAN + Supel™ QuE citrate/sodium bicarbonate	dSPE using Supel™ QuE PSA/C18 cleanup tube	LC-MS/MS: In MRM. ESI + and -	Zorbax SB-C18 (2.1 × 150 mm; 3.5 µm)	Gradient mode: 10 mM ammonium acetate solution in water [phase A] and ACN with 0.1% FA [B] at 400 µL/min	-	0.0005 - 0.002 mg/kg	[67]
Honey	2 g		QuEChERS	10 ml ACN + 4 g anhydrous $MgSO_4$ + 1 g trisodium citrate dihydrate + 0.5 g disodium hydrogen citrate sesquihydrate + 1 g NaCl	dSPE clean-up with 900 mg anhydrous $MgSO_4$ + 150 mg of PSA	GC-MS/MS: In SRM mode. EI ionization.	HP-5MS UI (15 m × 0.25 mm; 0.2 5 µm)	Helium	-	0.002 mg/kg	
Tomato	10 g	21	QuEChERS	10 ml ACN + 1 g NaCl + 1.5 g citrate	dSPE clean-up with 900 mg $MgSO_4$ + 150 mg PSA 150 mg C_{18}	OPPs: GC-NPD	HP-5MS column	-	72 - 116%	0.5 - 10 µg/kg	[72]
						Halogenated: GC-ECD	Quantification: DB-1 ; Confirmation: DB-17	-			
Lettuce						Methyl-carbamates: HPLC-FLD	Column waters	-			
						Imidacloprid and carbendazim: HPLC-DAD	Xterra RP-18	-			

96 types of vegetables	50 g	283	SLE	100 ml ACN; 10 g NaCl	For GC:<br>Sep-Pak Florisil (6cm3/1000mg) eluted with 7 ml 20% acetone/hexane<br><br>For LC:<br>Sep-Pak NH2 (6cm3/1000mg) eluted with 5 ml 1% MeOH/DCM	OPPs and nitrogen-containing compounds: GC-NPD<br>OCPs, dicarboximide and PYR: GC-μECD<br>Carbamate pesticides: LC-FLD<br>UV-detected compounds: LC-DAD: APCI +	HP-1701 (30m x 0.32mm; 0.25 μm)<br>HP-5 (30m x 0.32mm; 0.25 μm)<br>Carbamate analysis column (from Waters) (3.9 x 150 mm, 4 μm)<br>HP Zorbax Eclipse XDB-C 18 (250 x 4.6mm; 5 μm)	Nitrogen at 1400 μL/min, hydrogen at 3500 μL/min and air at 60000 μL/min<br>Nitrogen at 1000 μL/min<br>Gradient mode: 12% MeOH [phase A] MeOH-ACN-Water (35-35-30, v/v/v) [B] at 1000 μL/min<br>Gradient mode: 90% ACN [phase A] and water [B] at 1000 μL/min	82.5 - 103.1 %	0.0006 - 0.024 mg/kg	[65]
Tomato<br>French beans<br>Kale	10 g	7	QuEChERS	10 ml ACN + 150 mg $MgSO_4$	dSPE with 150 mg $MgSO_4$ + 50 mg PSA + 50 mg GCB	LC-MS/MS: ESI	Zorbax Eclipse Plus C18 (2.1 × 100 mm; 3.5 μm)	Gradient mode: 10mM ammonium formate in MeOH:H_2O (1:9 v/v) [phase A] and MeOH [B] at 300 μL/min	76.84 - 96.32 %	0.10 μg/kg	[73]
Tomato	10 g	2	QuEChERS	10 ml ethyl acetate + 4 g anhydrous $MgSO_4$ + 1 g NaCl	dSPE 50 mg PSA + 150 mg anhydrous $MgSO_4$	GC- ECD	DB 5 (30 m × 0.25 mm; 0.2 μm)	Helium at 1500 μL/min	83.1 - 102.2 %	0.01 mg/kg	[74]
Water spinach	5 g	2	SLE	10 ml ACN; 1.5 g NaCl	-	LC-MS/MS. ESI<br>Chlorothalonil: GC-MS: In SRM mode. EI	Agilent Poroshell 120 EC-C18 (3.0 mm × 50 mm; 2.7 μm)<br>Agilent HP-5 (30 m × 0.25 mm; 0.25 μm)	Gradient mode: 80% ACN [phase A] and 20% water [B] containing 0.2% (v/v) FA) at 500 μL/min<br>Helium at 3000 μL/min	91 - 101%<br>94 - 105%	0.02 mg/kg*<br>0.01 mg/kg *	[75]
Kale	10 g	4	SLE	15 ml MeOH	Purified with 50 mg C18	LC-MS/MS-QqQ: In MRM mode. ESI + and -	Zorbax XDB-C18 (4.6 × 150 mm; 5μm)	Gradient mode: ultrapure water [phase A] and MeOH [B] at 800 μL/min	26.5 - 89.6 %	0.14 - 20. 3 μg/kg	[76]
Apple	10 g	3	QuEChERS	10 ml ACN + 1 g NaCl + 4 g $MgSO_4$	dSPE with 250 mg $MgSO_4$ + 100 mg PSA + 15 mg GCB	Lambda-cyhalothrin: GC/MS: In SIM mode. EI<br>Thiamethoxam and clothianidin: RRLC-	J & W HP 5 (30 m x 0.25 mm; 0.25 μm)<br>ZORBAX Eclipse Plus Rapid	Helium at 1000 μL/min.<br>Gradient mode: FA in water (0.2%, v/v)	88 - 105%	0.01 mg/kg*	[77]

						MS/MS-QqQ: In MRM. EI +	Resolution C18 (2.1 mm i.d. × 50 mm; 1.8 µm)	[phase A] and ACN [B] at 300 µL/min			
Peaches	10 g	2	QuEChERS	20 ml ACN + 3 g NaCl	dSPE with 100 mg C18, 100 mg PSA + 300 mg of $MgSO_4$	LC-MS/MS-QqQ. ESI	Agilent Poroshell 120 EC-C18 (100 mm × 3 mm; 2.7 µm)	Gradient mode: MeOH [phase A] and 0.1% FA [B] at 400 µL/min	83 - 119%	0.01 mg/kg	[78]
Lettuce<br>Spinach<br>Spring onions<br>Peanuts	10 g<br><br><br>5 g	18	QuEChERS	10 ml ACN:AA (99:1, v/v) + 6 g $MgSO_4$ + 1.5 g NaOAc + 1.0 g sodium acetate trihydrate ($CH_3COONa·3H_2O$);	d-SPE clean-up with 1.2 g $MgSO_4$, 0.4 g C-18, 0.4 g PSA + 0.4 g Florisil	GC X GC-TOF-MS	Restek Rxi-5Sil MS (30 m × 0.25 mm; 0.25 µm) primary column coupled to an Rxi-17Sil MS (1.1 m × 0.25 mm; 0.25 µm) secondary column	Helium at 1400 µL/min	-<br>74 - 106%<br>-<br>73 - 101%	0.5 - 0.9 ng/g	[79]
Lettuce<br>Tomatoes<br>Cauliflower<br>Broad beans	2 g	8	SLE	10 ml ACN + 4g Na_2SO_4 +1 g NaCl	Two SPE purification: 1) d-SPE clean-up with 75 mg of C18, 75 mg of PSA + 1350 mg of Na_2SO_4; 2) SPE cartridges of 6cc/100mg eluted with 4 ml ethyl acetate	GC-MS/MS-QqQ: in SRM mode. EI	Sapiens X5-MS (20 m × 0.18 mm; 0.18 µm)	Helium at 600 µL/min	-<br>69 - 96 %<br>47 - 87 %<br>41 - 98 %	0.013 - 4.45 µg/kg	[80]
Wheat grain<br>Wheat straw	5 g<br>1 g	2	QuEChERS	10 ml ACN + 1 g NaCl + 4 g $MgSO_4$	d-SPE clean-up with 150 mg $MgSO_4$ + 50 mg C18+ 10 mg GCB	HPLC-MS/MS: in MRM mode	Agilent EC-C18 (50 mm × 3 mm; 2.7 µm)	Gradient mode: ACN [phase A] and 0.1% FA [B] 80:25 (v/v) at 300 µL/min	87 - 112% (epoxiconazole) and 85-102% (pyraclostrobin)	0.01 mg/kg*	[81]
Maize grain<br>Maize corncob<br>Maize straw	5 g<br>2 g<br>1 g	2	QuEChERS	10 ml 5% AA-ACN + 1 g NaCl + 4 g $MgSO_4$	Two types of dSPE: <u>Maize grain and straw</u>: 50 mg PSA + 5 mg MWCNTs + 150 mg $MgSO_4$; <u>Corncob extract</u>: 50 mg PSA + 150 mg $MgSO_4$	HPLC-MS/MS: in MRM mode. ESI -	Agilent ZORBAX SB-C18 (3.0 x 50 mm; 2.7 µm)	Gradient with ACN [phase A] and 0.2% FA [B] 60:40 (v/v) at 400 µL/min	98 - 107% (tembotrione) and 90-108% (M5)	0.43 - 1.5 µg/L	[82]
Soybean<br>Green soybean	5 g<br>5 g	5	QuEChERS	10 ml 1% AA-ACN + 1 g NaCl + 3 g $MgSO_4$	Two types of dSPE: <u>Soybean</u>: 50 mg	UPLC-QqQ-MS/MS. ESI	Agilent Poroshell 120 EC-C18	Isocratic mode: mobile phase (75% ACN + 25% water	71 - 116%	0.018 - 0.125 µg/kg	[83]

Soybean straw	2.5 g				C_{18} + 150 mg $MgSO_4$; <u>Green soybean and straw</u>: same + 5 mg MWCNTs		(50 × 3.0 mm; 2.7 µm)	10mM ammonium acetate) at 350 µL/min			
Common food (vegetables, fruit, cakes.)	10 g (non-cereal based).<br><br>5 g (cereal based)	516	Non cereal Based: QuEChERS (vers. 1);<br><br>Cereal Based: QuEChERS (vers. 2)	<u>Vers. 1</u>: 10 ml ACN + 1 g NaCl + 4 g $MgSO_4$ + 0.5 g disodium hydrogen citrate sesquihydrate + 1 g trisodium citrate dihydrate<br><br><u>Vers. 2</u>: 20 ml ACN + 1 g NaCl + 4 g MgSO4 + 0.5 g disodium hydrogen citrate sesquihydrate + 1 g trisodium citrate dihydrate;	dSPE: 150 mg MgSO4 + 25 mg PSA	221 analytes: LC-MS/MS-QqQ: ESI + and -	Water column (0.25 mm; 0.17 mm i. d)	Gradient mode: ammonium acetate (5 mM in water) [phase A] and MeOH [B] at 500 µL/min	70 - 120%	0.1-10 µg/kg	[66]
Baby food (prepared)			Non cereal based: QuEChERS (vers. 3);<br><br>Cereal Based: QuEChERS (vers. 4)	Vers. 3: 10 ml ethyl acetate + 1 g NaCl + 4 g $MgSO_4$ + 0.5 g disodium hydrogen citrate sesquihydrate + 1 g trisodium citrate dihydrate<br><br>Vers 4: 20 ml ethyl acetate:cyclohexane 81:1 + 1 g NaCl + 4 g $MgSO_4$ + 0.5 g disodium hydrogen citrate sesquihydrate + 1 g trisodium citrate dihydrated	Vers.3: Purified in HPGPC column<br><br>Vers 4: dSPE with 25 mg PSA + 25 mg C18 + 5 mg carbon	135 analytes: GC-MS/MS: in MRM mode. EI	Thermo Scientific TR-Pesticide II capillary column (30 m x 0.25 mm; 0.25 µm)	-			

µECD: micro-electron capture detector; AA: acetic acid; ACN: Acetonitrile; APCI: atmospheric pressure chemical ionization; DAD: diode array detection; DAI: direct aqueous injection; DCM: dichloromethane; ECD: electron capture detection; EI: electron ionization; ESI: electrospray ionization; FA: formic acid; FLD: fluorescence detector; GCB: graphitized carbon black; GFF: glass fiber filter; LLE: liquid-liquid extraction; MRM: multiple single reaction monitoring; MWCNTs: multiwalled carbon nanotubes; NaOAc: sodium acetate; NCI: negative chemical ionization; NPD: nitrogen–phosphorus detector; OCPs: Organochlorine Pesticides; OPPs: organophosphorus pesticides; PM: Particulate matter; PSA: primary secondary amines; PLE: pressurized liquid extraction; PUF: polyurethane foam; PYR: pyrethroid; SIM: selected ion monitoring; SLE: solid-liquid extraction SPE: solid phase extraction; SRM: selected reaction monitoring; TRV: toxicological reference value; Water: surface water

Table S2. Detailed information about the analytical methods published between 2019 and 2021 for the analysis of pesticides in biological matrices and between 2018 and 2021 for the Wastewater-Based Epidemiology (WBE) studies to analyse human exposure to pesticide residues.

**Sample**		**Nº Pesticides or biomarkers**	**Extraction**			**Separation and detection**			**Recovery %**	**LOD (or LOQ*)**	**Ref.**
**Matrix**	**Volume/ Weight**		**Method**	**Pretreatment/other features**	**Extraction/Clean-up**	**Technique**	**Separation equipment**	**Mobile phases**			
Urine	3 ml	5	LLE	Enzymatic digestion prior to extraction (175 µL β-glucuronidase enzyme + 125 µL 0.1 mol/L HAC–NaAC buffer)	2 ml ethyl acetate	HPLC-MS/MS-QqQ: in MRM. ESI +	Zorbax SB-C18 column (100 × 2.1 mm; 3.5µm)	Gradient mode: ACN [phase A] and 0.1% FA in water [B] at 400 µL/min	71 - 107 %	0.005 - 0.02 ng/ml*	[98]
Urine	4 ml	26	LLE: Dialkyl phosphates (DAPs)<br>QuEChERS: Specific metabolites	Digestion prior to extraction (800 µL HCl 6 M)<br>Enzymatic digestion prior to extraction (10 µL of β-glucuronidase aryl sulfatase enzyme)	4 ml ethyl acetate + 4ml diethyl ether<br>10 ml ACN	LC-MS/MS: in SRM. ESI (-) and APCI (-)	Symmetry C18 (2.1 × 150 mm; 5 µm)	Gradient mode: water [phase A] and MeOH [B] at 300 µL/min	70 - 120%	0.25 - 0.50 ng/ml*	[99]
Urine	2 ml	6	LLE	-	1º LLE: 2 mL ACN + 2 mL diethyl ether 2º LLE extraction: 5 ml water + 5 ml n-hexane	GC-MS/MS. EI +	HP-35MS (60 m x 0.25mm; 0.25µm)	Helium 5.0 at 1500µL/min	92 - 118%	0.01 - 0.1 ng/ml	[100]
Urine	2 ml	11	LLE	-	2 ml ACN + 2 ml diethyl ether	GC-MS: in MRM. EI +	DB-5MS (15m x 0.25mm; 0.25µm)	Methane	75 - 100%	0.1 - 0.5 ng/ml	[101]
Urine	1 ml	9	SPE	Enzymatic digestion prior to extraction (250µL of β-glucuronidase enzyme in ammonium acetate buffer [10mM, pH 6.7])	OASIS HLB (6cc/150mg) eluted with 6 ml MeOH	LC-MS/MS: in MRM. ESI -	BEH C18 column (100 x 2.1 mm; 1.7 µm)	Gradient mode: 0.1% FA in water [phase A] and ACN [B] at 350 µL/min	43 - 100%	0.00 1 - 0.3 ng/ml	[102]
Urine	1 ml	3	SPE	Enzymatic digestion prior to extraction (750µL of β-glucuronidase buffer solution)	OASIS HLB (6cc/150mg) eluted with 750 µL acetone	HPLC-MS/MS-QqQ: ESI + and -	Betasil C18 (100 mm × 2.1 mm; 3µm)	Gradient mode: 5% (v/v) MeOH in 1% (v/v) AA [phase A] and 100% ACN [B] at 500 µL/min	80-120%	0.003 - 0.4 ng/ml	[90]
Urine	4 ml	26	LLE: Dialkyl	Digestion prior to extraction (800 µL HCl 6M)	4 ml ethyl acetate + 4 ml diethyl ether	LC-MS/MS: in SRM. ESI -	Hypersil GOLD HILIC	Gradient mode: ammonium acetate 200mM in	82 - 117%	0.125 - 5.0 ng/ml*	[103]

			phosphates (DAPs)				(2.1 × 100 mm; 3 µm)	water [phase A] and in ACN [B] at 200 µL/min			
	5 ml		QuEChERS: Specific metabolites	Enzymatic digestion prior to extraction (10 µL of β-glucuronidase aryl sulfatase enzyme)	10 ml ACN				60 - 120%		
Urine	0.1 ml	2	LLE	-	1 ml ACN	GC-MS/MS: ESI -	HP INNOWax (30 m × 0.25 mm; 0.25 column µm)	Helium 4.5 at 1200 µL/min	90 - 110%	0.1 ng/ml*	[89]
Urine	1 ml	3	LLE	-	1 ml ethyl acetate	UPLC-MS/MS: in MRM. ESI -	Atlantis T3 (150 mm × 2.1 mm; 5 µm)	Gradient mode: 0.1% FA [phase A] and MeOH with 5% A (v/V) [B] at 200µL/min	86 - 108%	0.02 - 0.09 ng/ml	[104]
Urine	4 ml	43	SPE: Parent pesticides and desethylterbuthylazine	Enzymatic digestion prior to extraction (20 µL of β-glucuronidase enzyme + 20 µL sulfatase + 2 ml 0.2M sodium acetate buffer)	Oasis HLB (6cc/200mg) + Chromafix Dry sodium sulfate cartridge eluted with	GC-MS/MS-QqQ: in MRM. PCI -	HP5-MS (30m x 0.25 mm; 0.25 µm)	Helium at 1200 µL/min	-	0.05 - 0.92 ng/ml*	[63]
				Enzymatic digestion prior to extraction (20 µL of β-glucuronidase enzyme + 20 µL sulfatase + 2 ml 0.2M sodium acetate buffer)	Oasis HLB (6cc/200mg) eluted with 3 ml DCM	LC-MS/MS: in MRM. ESI +	UPLC BEH C18 (100 x 2.1 mm; 1.7 µm)	Gradient mode: 0.1% FA [phase A] and ACN [B] at 20 µL/min	-		
	2 ml		SPE: DAPs	Digestion prior to extraction (300 µL HCA 3M);	Oasis WAX (3cc/60mg) eluted with 5% ammoniac in MeOH	GC-MS-QqQ: in MRM. PCI -	HP5-MS (30m x 0.25 mm; 0.25 µm)	Helium at 1000 µL/min	-		
	3 ml		LLE: Pyrethroid metabolites and other biomarkers	Enzymatic digestion prior to extraction (20 µL of β-glucuronidase enzyme + 20 µL sulfatase + 2 ml 0.2M sodium acetate buffer)	4 ml diethyl ether + 2 ml sodium dihydrogen phosphate (0.2M)	GC-MS-QqQ: in MRM. EI +	HP5-MS (30m x 0.25 mm; 0.25 µm)	Helium at 1200 µL/min	-		
Urine	3 ml	6	LLE	Enzymatic digestion prior to extraction (0.3 ml 1.0 M ammonium acetate with 66 units of β-glucuronidase enzyme)	4 ml ethyl acetate	HPLC-MS/MS: in MRM mode. ESI +	Atlantis®d-C18 (150 x 2.1 mm; 5 µm)	Gradient mode: 0.1% FA in water [phase A] and ACN [B] at 600 µL/min	76 - 107%	0.0002-0.006 ng/mL	[105]
Urine	1 ml	2	SPE	Enzymatic digestion prior to extraction (10 µL IS solution [1 mg/L] and 100	PEP cartridges eluted with 1 ml ACN	LC-MS/MS: in MRM mode. ESI +	C18 (2.1 mm × 50 mm; 1.7 µm)	Gradient mode: MeOH [phase A] and MilliQ water	78- 111%	0.029 - 0.038 ng/ml*	[106]

				µL β-glucuronidase enzyme [124 units/ml] solution with ammonium acetate)				[B] bouth 0.2% FA at 300 µL/min			
Urine	0.5 ml	11	SPE	Enzymatic digestion prior to extraction (400 µL 0.2 M sodium acetate with 745 units/ml of β-glucuronidase enzyme and 56 units/ml of sulfatase)	Oasis® HLB (3 cc/60mg) eluted with 3 ml acetone + 3 ml hexane	HPLC-MS/MS-QqQ: ESI + and -	Betasil C18 (100 × 2.1 mm; 5 µm)	Gradient mode: 0.2% (v/v) AA [phase A] and ANC [B] at 350 µL/min	84-115%	0.025-0.05 ng/mL	[107]
Urine	1 ml	6	SPE	Enzymatic digestion prior to extraction (750 µL β-glucuronidase enzyme buffer solution)	Quadra 3 Liquid Handling Station and OASIS HLB 96-well (automated SPE) eluted with 750 µL acetone (in two 325 µL aliquots)	HPLC-MS-QqQ: in SRM mode. ESI + and -	Betasil C18 (100 mm × 2.1 mm; 3 µm)	Gradient mode: 5% (v/v) methanol in 1% (v/v) aqueous AA [phase A] and 100% ACN [B] at 500 µL/min	90 - 110%	0.1 - 0.5 µg/L	[108]
Urine	0.6 ml	12	-	Digestion prior to extraction (25 µL of 1.3% FA in water)	-	LC-MS/MS-QqQ: ESI	Bio-Rad Cation-H (30 mm × 4.6 mm)	Gradient mode: 0.1% FA in water [phase A] and ACN [B] with flow ramps	-	0.1 - 0.5 ng/mL	[109]
Urine	2 ml	6	LLE	-	2 ml ACN + 2 ml diethyl ether	GC-MS/MS-QqQ: EI	DB5-MS (30 m × 0.25 mm; 0.25 µm)	Helium at 1000 µL/min	76 - 110%	0.0032 - 0.31 ng/mL	[110]
Urine	2 ml	2	LLE	Digestion prior to extraction (0.5 mL concentrated HCl)	2 ml MTBE	GC-MS/MS-QqQ: in MRM mode. EI	Rxi–5 ms (30 m × 250 µm; 0.25 µm)	Helium at 1000 µL/min	91 - 109 %	0.049 - 0.075 µg/L	[111]
Urine	1 ml	7	SPE	-	Presep RPP cartridges (60 mg) + ENVIcarb/PSA (500 mg/300mg) eluted with 8 ml DCM:ACN (2:8; v/v)	LC-MS/MS: in MRM mode. ESI +	RSpak DE-213 (150 mm × 2 ID mm)	Gradient mode: 0.1% (v/v) FA 10mM AA in water [phase A] and 0.1% (v/v) FA 10mM AA in MeOH at 400 µL/min	96 - 102%	0.05 - 0.2 ng/mL*	[60]
Urine	0.3 ml	3	SPE	Digestion prior to extraction (40 µL FA)	Strata-X-AW eluted with0.5 ml acetone (5% TEA)	UHPLC-TOFMS: ESI -	Acquity UPLC® BEH C18 (100 mm × 2.1 mm; 1.7 µm)	Gradient mode: 99% H_2O with 0.5 mM HCOOH/TPAF [phase A] and 1%	42 - 108% (100 ng/ml)	0.05 - 3.03 µg/L	[93]

								MeCN [B] at 400 µL/min			
Blood	2 - 5 g	6	SPE	Digestion prior to extraction (5 ml FA:2-propanol (4:1, v/v) and diluted with 5 ml 10% 2-propanol in water)	ASPEC XL4 + Oasis © HLB (3cc/400mg) (Automated SPE)	GC-MS/MS-QqQ	DB-5MS UI (20 m × 0.18 mm; 0.18 µm)	Helium at 700 µL/min	-	0.3 - 1.52 pg/g	
Urine	5 ml		SPE	-	C-18 Sep-Pak cartridges (500 m eluted with 5 ml DCM	UHPLC-MS/MS-QqQ: in MRM mode. ESI +	Acquity UPLC™ BEH C18 (100 mm × 2.1 mm ; 1.7 µm)	Gradient mode: 0.01% FA in water [phase A] and MeOH [B] in 350 µl/min	60 - 120%	0.5 - 1.0 µg/L	
Hair (dried)	0.05 g	4	SLE	Incubated in ultrasonic bath (4h) with 2 ml MeOH prior to extraction	2 ml MeOH and cleaned up in Purified in an econofilter with 15 mg of K_2CO_3 + 50 mg $Na_2S_2O_5$ eluted with 1 ml ACN + 15 mg K_2CO_3 + 0.1 ml PFBBRr in ACN (1/3, v/v))	GC–MS: in SIM mode	BPX5 (30 m × 0.25 mm; 0.25 µm)	Helium at 1000 µl/min	75 - 107 %	3 - 6 pg/g	[88]
Urine	1 ml		SPE: 3-phenoxybenzoic acid (non-specific metabolite of PYR)	Enzymatic digestion prior to extraction (750 µL of β-glucuronidase buffer solution)	OASIS HLB (6cc/150mg) eluted with 750 µL acetone	HPLC-MS/MS-QqQ: ESI + and -	Betasil C18 (100 mm × 2.1 mm; 3µm)	Gradient mode: 5% (v/v) MeOH in 1% (v/v) AA [phase A] and 100% ACN [B] at 500 µL/min	80 - 120%		
	2 ml	11	LLE: Dialkyl phosphates (metabolites of OPPs)	-	2mL ACN + 2mL ethyl ether	GC-MS/MS: in MRM. ESI +	DB-5MS (30 m x 0.25mm; 0.25µm)	Methane	75 - 100%	0.015 - 4.0 ng/ml	[112]
Serum	4 g		SPE: OCPs	-	Oasis HLB (540mg) (Automated SPE workstation); Clean-up with two-layered SPE cartridge eluted	HRGC-IDHRMS: ESI -	DB5HT (15 m x 0.25 mm; 0.10 µm)	-	69 - 98%		

Serum	0.5 ml	26	SPE	-	with 12 ml DCM; 8 ml hexane Oasis HLB (6cc/500mg); Clean-up with a small multilayer silica gel column (2ml, 1.5g) 10 ml n-hexane; 7.5 ml hexane	HRGC-HRMS: EI +	DB-5MS (30 m x 0.25 mm; 0.25 μm)	Helium at 1000 μL/min	30 - 124%	0.07 - 13.44 pg/ml	[113]
Serum	0.250 ml	31	SPE	Enzymatic digestion prior to extraction (400 μL 0.2M sodium acetate buffer with 745 units/mL of β-glucuronidase enzyme + 56 units/ml of sulfatase)	Oasis HLB (3cc/60mg) eluted with 3ml acetone + 3ml hexane	HPLC-MS/MS: ESI +	Betasil C18 (100 × 2.1 mm; 5 μm) serially connected to a Javelin Betasil C18 pre-column (20 × 2.1 mm; 5 μm)	Gradient mode: ACN [phase A] and 0.2% AA [B] at 350 μL/min	80 - 119%	0.001 - 1.46 ng/ml	[114]
Serum	5 - 10 ml (collected)	8	SPE	-	Sepra C18-E with Silica gel/Sulfuric Acid (2:1 w/w)	HRGC-HRMS: in SIM mode	DB5HT (15 m x 0.25-mm; 0.10 μm)	-	-	0.001 - 0.005 ng/mL	[115]
Serum	1 g	11	PLE	Dried serum in extraction cells with 3g hydromatrix	20% DCM in hexane	GC-HRMS: EI	DB-5MS (30 m×0.25 mm; 0.25 μm)	Helium at 1000 μL/min	-	5 pg/g	[92]
Serum	2 g	9	LLE	Digestion prior to extraction (0.5 ml 6M HCl)	2.5 ml isopropanol + 6 ml of 50% MTBE in hexane Two cleanup cartridge: Top: 0.2 g silica gel; Lower: 1.0 g 33% sulfuric acid in silica gel (v/v) eluted with 10 ml 5% DCM in hexane	GC-IDHRMS	-	-	64 - 74%	11.5 ng/g	[116]
Plasma	0.5 ml	1	LLE	Digestion prior extraction (2 ml FA [50% v/v])	5 ml hexane; Purified with ISOLUTE Florisil cartridges: 0.5 g anhydrous sodium sulfate + 1.8 g acidified silica eluted with 20 ml	GC-MS/MS: in MRM mode. EI	ZB-5HT (15 m × 0.250; 0.10 μm)	Helium at 2250 μL/min	-	0.01 ng/ml	[91]

Breast Milk	2 ml	18	LLE	-	hexane:DCM (19:1 v/v) First LLE: 15 ml hexane:acetone (1:1); Second LLE: 10ml hexane:acetone; Third LLE: 15ml sodium sulfate 2% + 10ml hexane + 5ml acetone	GC-MS	DB-5 (30 m x 0.25 mm; 25 μm)	Helium at 1000 μL/min	60 - 120%	1.7 - 4.3 ng/g	[84]
Breast Milk	10 ml	3	QuEChERS	-	20 ml ACN + 4g anhydrous $MgSO_4$ + 1.5 g anhydrous NaCl; d-SPE clean-up with 50 mg PSA + 50mg C18 + 750 mg $MgSO_4$	Analyzed with GC-ECD; Confirmed with GC-MS	GC-ECD: DB-1 (30m x 0.25 mm; 0.25 μm); GC-MS: DB-5 (30mx 0.25mm; 0.25 μm)	Nitrogen at 30000 μL/min (GC-ECD); Helium (GC-MS)	85.8 - 120%	0.005 - 0.05 mg/kg	[85]
Breast Milk	10 ml	161	QuEChERS	-	10 ml ACN + 4 g $MgSO_4$ + 1g NaCl + 1g sodium dibasic citrate + 0.5g sodium tribasic citrate; Clean-up in Polish EMR lipid tube ($MgSO_4$ + NaCl)	LC- MS/MS: in MRM mode. ESI	C18 (150 x 2.1 mm; 3.5μm)	Gradient mode: 5mM ammonium acetate and 0.1% FA in water [phase A] and 5 mM ammonium acetate in MeOH [B] at 400 μl/min	80 - 120%	5 μg/kg*	[86]
Breast milk<br>Maternal Blood<br>Placenta Cord blood	1 - 5 g	28	LLE	-	5 ml cyclohexane:acetone (3:2); One aliquot + 97.5% H_2SO_4 One aliquot + GPC	HRGC-ECD<br>HRGC-LRMS: in SIM mode. ECNI	1 m long re-column connect to a dual column system: SPB-E and SPB-1701 (60 m x 0.25 mm; 0.25 μm)<br>DB-5 MS (60 m x 0.25 mm; 0.25 μm)	Hydrogen at 50000<br>Helium at 1000 μl/min	77 - 159%<br>104 - 161%	0.002 - 0.041 ng/g<br>0.014-0.626 ng/g	[87]
Untreated wastewater	50 mL	14	Alkylphosphates: direct	Final extract reconstituted in 100 μL MilliQ-Water	OASIS® HLB (3cc/60 mg) eluted with 3 ml MeOH	LC-QqQ-MS/MS: in SRM. Ionization + and -	XSELECT™ CSH™ C18 (100 x 2.1 mm; 2.5 μm)	Gradient mode: 0.1 % AA in water [phase A] and	80 - 120%	0.30 – 474 ng/L	[46]

			injection; Others: SPE					acetonitrile [B] at 180 µL/min			
Untreated wastewater	50 mL	9	SPE	Final extract reconstituted in 500 µL 80:20 H_2O:MeOH	OASIS® HLB (3cc/60 mg) eluted with 4 ml MeOH	UHPLC-QqQ-MS/MS: in MRM. Ionization + and -	BEH C18 (150 × 1.0 mm; 1.7 µm)	ESI-: gradient mode: 1mM NH_4F 80:20 H_2O: MeOH [phase A] and 1mM NH_4F 5:95 H_2O:MeOH [B]. ESI +: gradient mode: 5mM NH_4OAc 80:20 H_2O:MeOH [A] and 100% MeOH [B]. At 40 µL/min	80 - 120%	0.02 - 0.95 ng/L	[45]
Untreated wastewater	50 mL	18	SPE	Final extract reconstituted in 100 µL of MilliQ-Water	OASIS ® HLB (3cc/60 mg) eluted with 3 ml MeOH	LC-MS/MS-QqQ: in SRM mode. Ionization in + and -	XSELECT™ CSH™ C18 (100 x 2.1 mm; 2.5 µm)	Gradient mode: 0.1% AA in water [phase A] and in ACN [B] at 180 µL/min	75 - 115%	1.0 - 790 ng/L	[47]
Untreated wastewater	100 mL	3	LLE	Final extract 1ml hexane/acetane concentrated to 0.5 ml	30 ml hexane:acetane (85:15, v/v)	GC-QqQ-MS/MS: Ionization in +	Rxi-1MS (30 m x 0.25 mm; 0.25 µm)	Carrier gas at 1000 µL/min	80 - 120%	100 ng/L	[48]

AA: Acetic acid; ACN: acetonitrile; ASE: accelerated solvent extraction; BSTFA: N,O-Bis(trimethylsilyl)trifluoroacetamide; CIP: chloroiodopropane; DAP: dialkyl phosphates; DCM: dichloromethane; ECNI: electron capture negative ionization; EMR: enhanced Matrix Removal; ESI: electrospray ionization; FA: formic acid; GPC: gel permeation column; HAC-NaAc: acetic acid-sodium acetate; HCA: hydrochloric acid; ID: internal diameter; LLE: liquid-liquid extraction; MRM: multiple reaction monitoring; MTBE: methyl tert-butyl ether; MTBSTFA: N-tert-Butyldimethylsilyl-N-methyltrifluoroacetamide; NH4OAc: Ammonium Acetate; OCPs: organochloride pesticides; OPPs: organophosphate pesticides; PFBBr: pentafluorobenzyl bromide; PLE: pressurized liquid extraction; PYR: pyrethroids; SIM: selected ion monitoring; SLE: solid-liquid extraction; SPE: solid phase extraction; SRM: selected reaction monitoring; TEA: triethylamine; TPAF: tripropylammonium formate

References

References follow the same order as the main manuscript.

1. Hassaan, M.A.; El Nemr, A. Pesticides Pollution: Classifications, Human Health Impact, Extraction and Treatment Techniques. *The Egyptian Journal of Aquatic Research* **2020**, *46*, 207–220, doi:10.1016/j.ejar.2020.08.007.
2. Jia, M.; E, Z.; Zhai, F.; Bing, X. Rapid Multi-Residue Detection Methods for Pesticides and Veterinary Drugs. *Molecules* **2020**, *25*, 3590, doi:10.3390/molecules25163590.
3. Food And Agriculture Organization Of The United Nations *The International Code of Conduct on Pesticide Management*; Food & Agriculture Org, 2015; ISBN 92-5-408548-0.
4. Yadav, I.C.; Devi, N.L.; Syed, J.H.; Cheng, Z.; Li, J.; Zhang, G.; Jones, K.C. Current Status of Persistent Organic Pesticides Residues in Air, Water, and Soil, and Their Possible Effect on Neighboring Countries: A Comprehensive Review of India. *Science of The Total Environment* **2015**, *511*, 123–137, doi:10.1016/j.scitotenv.2014.12.041.
5. Narenderan, S.T.; Meyyanathan, S.N.; Babu, B. Review of Pesticide Residue Analysis in Fruits and Vegetables. Pre-Treatment, Extraction and Detection Techniques. *Food Research International* **2020**, *133*, 109141, doi:10.1016/j.foodres.2020.109141.
6. Yadav, I.C.; Devi, N.L. Pesticides Classification and Its Impact on Human and Environment. *Environmental science and engineering* **2017**, *6*, 140–158.
7. Chandran, C.S.; Thomas, S.; Unni, M. Pesticides: classification, detection, and degradation. In *Organic Farming*; Springer, 2019; pp. 71–87.
8. BINNS, J. EU Pesticides Database Available online: https://ec.europa.eu/food/plant/pesticides/eu-pesticides-db_en (accessed on 24 February 2021).
9. FAOSTAT Available online: http://www.fao.org/faostat/en/#data/RP/visualize (accessed on 16 February 2021).
10. Carriger, J.F.; Rand, G.M.; Gardinali, P.R.; Perry, W.B.; Tompkins, M.S.; Fernandez, A.M. Pesticides of Potential Ecological Concern in Sediment from South Florida Canals: An Ecological Risk Prioritization for Aquatic Arthropods. *Soil & Sediment Contamination* **2006**, *15*, 21–45.
11. Popp, J.; Pető, K.; Nagy, J. Pesticide Productivity and Food Security. A Review. *Agron. Sustain. Dev.* **2013**, *33*, 243–255, doi:10.1007/s13593-012-0105-x.
12. An, Y. Detection of Pesticide Residues in Soil, Water, and Food. *IOP Conf. Ser.: Earth Environ. Sci.* **2020**, *544*, 012009, doi:10.1088/1755-1315/544/1/012009.
13. Nasiri, M.; Ahmadzadeh, H.; Amiri, A. Sample Preparation and Extraction Methods for Pesticides in Aquatic Environments: A Review. *TrAC Trends in Analytical Chemistry* **2020**, *123*, 115772, doi:10.1016/j.trac.2019.115772.
14. Tian, D.; Mao, H.; Lv, H.; Zheng, Y.; Peng, C.; Hou, S. Novel Two-Tiered Approach of Ecological Risk Assessment for Pesticide Mixtures Based on Joint Effects. *Chemosphere* **2018**, *192*, 362–371, doi:10.1016/j.chemosphere.2017.11.001.
15. Wang, X.; Zhong, W.; Xiao, B.; Liu, Q.; Yang, L.; Covaci, A.; Zhu, L. Bioavailability and Biomagnification of Organophosphate Esters in the Food Web of Taihu Lake, China: Impacts of Chemical Properties and Metabolism. *Environment International* **2019**, *125*, 25–32, doi:10.1016/j.envint.2019.01.018.
16. Jeyaratnam, J. Acute Pesticide Poisoning: A Major Global Health Problem. *World health statistics quarterly 1990; 43 (3): 139-144* **1990**.
17. WHO \textbar Acute Pesticide Poisoning: A Proposed Classification Tool Available online: https://www.who.int/bulletin/volumes/86/3/07-041814/en/ (accessed on 22 February 2021).
18. Rani, L.; Thapa, K.; Kanojia, N.; Sharma, N.; Singh, S.; Grewal, A.S.; Srivastav, A.L.; Kaushal, J. An Extensive Review on the Consequences of Chemical Pesticides on Human Health and Environment. *Journal of Cleaner Production* **2021**, *283*, 124657, doi:10.1016/j.jclepro.2020.124657.
19. *Directive 2006/11/EC of the European Parliament and of the Council of 15 February 2006 on Pollution Caused by Certain Dangerous Substances Discharged into the Aquatic Environment of the Community (Codified Version)* **2006**.
20. *Regulation (EU) 2019/1009 of the European Parliament and of the Council of 5 June 2019 Laying down Rules on the Making Available on the Market of EU Fertilising Products and Amending Regulations (EC) No 1069/2009 and (EC) No 1107/2009 and Repealing Regulation (EC) No 2003/2003,* **2019**, Vol. 170.
21. *Regulation (EU) No. 528/2012 of the European Parliament and of the Council of 22 May 2012 Concerning the Making Available on the Market and Use of Biocidal Products. Official Journal of the European Union* **2012**, Vol 167.
22. R*egulation (EC) No 396/2005 of the European Parliament and of the Council of 23 February 2005 on Maximum Residue Levels of Pesticides in or on Food and Feed of Plant and Animal Origin and Amending Council Directive 91/414/EEC*, **2012**.
23. *Directive 2009/128/EC of the European Parliament and of the Council of 21 October 2009 Establishing a Framework for Community Action to Achieve the Sustainable Use of Pesticides. Official Journal of the European Union* **2009**, Vol 309.
24. N. Kkkinakis, M.; N. Tzatzarakis, M.; Tsakiris, I.; I. Vardavas, A.; I. Vardavas, C.; Stivaktakis, P.; Kokkinaki, A. Pesticide Residue Monitoring in the EuropeanUnion Agricultural Sector via Modern Analyticaltechniques.A

Review on Organophosphates. *International Journal of Biology and Biomedical Engineering* **2020**, *14*, doi:10.46300/91011.2020.14.23.
25. Teysseire, R.; Manangama, G.; Baldi, I.; Carles, C.; Brochard, P.; Bedos, C.; Delva, F. Determinants of Non-Dietary Exposure to Agricultural Pesticides in Populations Living Close to Fields: A Systematic Review. *Science of The Total Environment* **2021**, *761*, 143294, doi:10.1016/j.scitotenv.2020.143294.
26. Silva Pinto, B.G.; Marques Soares, T.K.; Azevedo Linhares, M.; Castilhos Ghisi, N. Occupational Exposure to Pesticides: Genetic Danger to Farmworkers and Manufacturing Workers – A Meta-Analytical Review. *Science of The Total Environment* **2020**, *748*, 141382, doi:10.1016/j.scitotenv.2020.141382.
27. Ohlander, J.; Fuhrimann, S.; Basinas, I.; Cherrie, J.W.; Galea, K.S.; Povey, A.C.; van Tongeren, M.; Harding, A.-H.; Jones, K.; Vermeulen, R.; et al. Systematic Review of Methods Used to Assess Exposure to Pesticides in Occupational Epidemiology Studies, 1993–2017. *Occup Environ Med* **2020**, *77*, 357–367, doi:10.1136/oemed-2019-105880.
28. Katsikantami, I.; Colosio, C.; Alegakis, A.; Tzatzarakis, M.N.; Vakonaki, E.; Rizos, A.K.; Sarigiannis, D.A.; Tsatsakis, A.M. Estimation of Daily Intake and Risk Assessment of Organophosphorus Pesticides Based on Biomonitoring Data – The Internal Exposure Approach. *Food and Chemical Toxicology* **2019**, *123*, 57–71, doi:10.1016/j.fct.2018.10.047.
29. Dereumeaux, C.; Fillol, C.; Quenel, P.; Denys, S. Pesticide Exposures for Residents Living Close to Agricultural Lands: A Review. *Environment International* **2020**, *134*, 105210, doi:10.1016/j.envint.2019.105210.
30. Sagiv, S.K.; Harris, M.H.; Gunier, R.B.; Kogut, K.R.; Harley, K.G.; Deardorff, J.; Bradman, A.; Holland, N.; Eskenazi, B. Prenatal Organophosphate Pesticide Exposure and Traits Related to Autism Spectrum Disorders in a Population Living in Proximity to Agriculture. *Environmental health perspectives* **2018**, *126*, 047012.
31. Goodman, J.E.; Prueitt, R.L.; Boffetta, P.; Halsall, C.; Sweetman, A. "Good Epidemiology Practice" Guidelines for Pesticide Exposure Assessment. *International Journal of Environmental Research and Public Health* **2020**, *17*, 5114, doi:10.3390/ijerph17145114.
32. Ring, C.L.; Arnot, J.A.; Bennett, D.H.; Egeghy, P.P.; Fantke, P.; Huang, L.; Isaacs, K.K.; Jolliet, O.; Phillips, K.A.; Price, P.S.; et al. Consensus Modeling of Median Chemical Intake for the U.S. Population Based on Predictions of Exposure Pathways. *Environ. Sci. Technol.* **2019**, *53*, 719–732, doi:10.1021/acs.est.8b04056.
33. Melymuk, L.; Demirtepe, H.; Jílková, S.R. Indoor Dust and Associated Chemical Exposures. *Current Opinion in Environmental Science & Health* **2020**, *15*, 1–6, doi:10.1016/j.coesh.2020.01.005.
34. Anh, H.Q.; Tran, T.M.; Thu Thuy, N.T.; Minh, T.B.; Takahashi, S. Screening Analysis of Organic Micro-Pollutants in Road Dusts from Some Areas in Northern Vietnam: A Preliminary Investigation on Contamination Status, Potential Sources, Human Exposure, and Ecological Risk. *Chemosphere* **2019**, *224*, 428–436, doi:10.1016/j.chemosphere.2019.02.177.
35. Bruce-Vanderpuije, P.; Megson, D.; Reiner, E.J.; Bradley, L.; Adu-Kumi, S.; Gardella, J.A. The State of POPs in Ghana- A Review on Persistent Organic Pollutants: Environmental and Human Exposure. *Environmental Pollution* **2019**, *245*, 331–342, doi:10.1016/j.envpol.2018.10.107.
36. Dalmolin, S.P.; Dreon, D.B.; Thiesen, F.V.; Dallegrave, E. Biomarkers of Occupational Exposure to Pesticides: Systematic Review of Insecticides. *Environmental Toxicology and Pharmacology* **2020**, *75*, 103304, doi:10.1016/j.etap.2019.103304.
37. Global Environment Monitoring System-Food Contamination Monitoring and Assessment Programme (GEMS/Food). *Guidelines for Predicting Dietary Intake of Pesticides Residues (Revised)*. World Health Organization: Switzerland, **2000**.
38. World Health Organization, Food and Agriculture Organization of the United Nations. *The International Code of Conduct on Pesticide Management*. FAO and WHO: Rome, **2014**.
39. Mungai, T.M.; Wang, J. Occurrence and Toxicological Risk Evaluation of Organochlorine Pesticides from Suburban Soils of Kenya. *International Journal of Environmental Research and Public Health* **2019**, *16*, 2937, doi:10.3390/ijerph16162937.
40. Tran, T.A.M.; Malarvannan, G.; Hoang, T.L.; Nguyen, V.H.; Covaci, A.; Elskens, M. Occurrence of Organochlorine Pesticides and Polychlorinated Biphenyls in Sediment and Fish in Cau Hai Lagoon of Central Vietnam: Human Health Risk Assessment. *Marine Pollution Bulletin* **2019**, *141*, 521–528, doi:10.1016/j.marpolbul.2019.03.006.
41. Ali, S.N.; Baqar, M.; Mumtaz, M.; Ashraf, U.; Anwar, M.N.; Qadir, A.; Ahmad, S.R.; Nizami, A.-S.; Jun, H. Organochlorine Pesticides in the Surrounding Soils of POPs Destruction Facility: Source Fingerprinting, Human Health, and Ecological Risks Assessment. *Environ Sci Pollut Res* **2020**, *27*, 7328–7340, doi:10.1007/s11356-019-07183-7.
42. Solomon, K.R. Estimated Exposure to Glyphosate in Humans via Environmental, Occupational, and Dietary Pathways: An Updated Review of the Scientific Literature. *Pest Manag Sci* **2020**, *76*, 2878–2885, doi:10.1002/ps.5717.
43. Chandra Yadav, I.; Devi, N.L.; Li, J.; Zhang, G. Polychlorinated Biphenyls and Organochlorines Pesticides in Indoor Dust: An Exploration of Sources and Health Exposure Risk in a Rural Area (Kopawa) of Nepal. *Ecotoxicology and Environmental Safety* **2020**, *195*, 110376, doi:10.1016/j.ecoenv.2020.110376.
44. Chandra Yadav, I.; Devi, N.L.; Li, J.; Zhang, G. Examining the Role of Total Organic Carbon and Black Carbon in the Fate of Legacy Persistent Organic Pollutants (POPs) in Indoor Dust from Nepal: Implication on Human Health. *Ecotoxicology and Environmental Safety* **2019**, *175*, 225–235, doi:10.1016/j.ecoenv.2019.03.048.

45. Kasprzyk-Hordern, B.; Proctor, K.; Jagadeesan, K.; Lopardo, L.; O'Daly, K.J.; Standerwick, R.; Barden, R. Estimation of Community-Wide Multi-Chemical Exposure via Water-Based Chemical Mining: Key Research Gaps Drawn from a Comprehensive Multi-Biomarker Multi-City Dataset. *Environment International* **2021**, *147*, 106331, doi:10.1016/j.envint.2020.106331.
46. Rousis, N.I.; Gracia-Lor, E.; Reid, M.J.; Baz-Lomba, J.A.; Ryu, Y.; Zuccato, E.; Thomas, K.V.; Castiglioni, S. Assessment of Human Exposure to Selected Pesticides in Norway by Wastewater Analysis. *Science of The Total Environment* **2020**, *723*, 138132, doi:10.1016/j.scitotenv.2020.138132.
47. Devault, D.A.; Karolak, S.; Lévi, Y.; Rousis, N.I.; Zuccato, E.; Castiglioni, S. Exposure of an Urban Population to Pesticides Assessed by Wastewater-Based Epidemiology in a Caribbean Island. *Science of The Total Environment* **2018**, *644*, 129–136, doi:10.1016/j.scitotenv.2018.06.250.
48. Devault, D.A.; Amalric, L.; Bristeau, S. Chlordecone Consumption Estimated by Sewage Epidemiology Approach for Health Policy Assessment. *Environ Sci Pollut Res* **2018**, *25*, 29633–29642, doi:10.1007/s11356-018-2995-x.
49. Huang, F.; Li, Z.; Zhang, C.; Habumugisha, T.; Liu, F.; Luo, X. Pesticides in the Typical Agricultural Groundwater in Songnen Plain, Northeast China: Occurrence, Spatial Distribution and Health Risks. *Environ Geochem Health* **2019**, *41*, 2681–2695, doi:10.1007/s10653-019-00331-5.
50. Dong, W.; Zhang, Y.; Quan, X. Health Risk Assessment of Heavy Metals and Pesticides: A Case Study in the Main Drinking Water Source in Dalian, China. *Chemosphere* **2020**, *242*, 125113, doi:10.1016/j.chemosphere.2019.125113.
51. Bradley, P.M.; Romanok, K.M.; Duncan, J.R.; Battaglin, W.A.; Clark, J.M.; Hladik, M.L.; Huffman, B.J.; Iwanowicz, L.R.; Journey, C.A.; Smalling, K.L. Exposure and Potential Effects of Pesticides and Pharmaceuticals in Protected Streams of the US National Park Service Southeast Region. *Science of The Total Environment* **2020**, *704*, 135431, doi:10.1016/j.scitotenv.2019.135431.
52. Lu, C.; Lu, Z.; Lin, S.; Dai, W.; Zhang, Q. Neonicotinoid Insecticides in the Drinking Water System – Fate, Transportation, and Their Contributions to the Overall Dietary Risks. *Environmental Pollution* **2020**, *258*, 113722, doi:10.1016/j.envpol.2019.113722.
53. Mahai, G.; Wan, Y.; Xia, W.; Wang, A.; Shi, L.; Qian, X.; He, Z.; Xu, S. A Nationwide Study of Occurrence and Exposure Assessment of Neonicotinoid Insecticides and Their Metabolites in Drinking Water of China. *Water Research* **2021**, *189*, 116630, doi:10.1016/j.watres.2020.116630.
54. Jin, H.; Dai, W.; Li, Y.; Hu, X.; Zhu, J.; Wu, P.; Wang, W.; Zhang, Q. Semi-Volatile Organic Compounds in Tap Water from Hangzhou, China: Influence of Pipe Material and Implication for Human Exposure. *Science of The Total Environment* **2019**, *677*, 671–678, doi:10.1016/j.scitotenv.2019.04.387.
55. Arisekar, U.; Jeya Shakila, R.; Shalini, R.; Jeyasekaran, G. Pesticides Contamination in the Thamirabarani, a Perennial River in Peninsular India: The First Report on Ecotoxicological and Human Health Risk Assessment. *Chemosphere* **2021**, *267*, 129251, doi:10.1016/j.chemosphere.2020.129251.
56. Bhandari, G.; Atreya, K.; Scheepers, P.T.J.; Geissen, V. Concentration and Distribution of Pesticide Residues in Soil: Non-Dietary Human Health Risk Assessment. *Chemosphere* **2020**, *253*, 126594, doi:10.1016/j.chemosphere.2020.126594.
57. Lehotay, S. AOAC Official Method 2007.01 Pesticide Residues in Foods by Acetonitrile Extraction and Partitioning with Magnesium Sulfate. *Journal of AOAC International* **2007**, *90*, 485–520.
58. Yera, A.; Nascimento, M.; da Rocha, G.; de Andrade, J.; Vasconcellos, P. Occurrence of Pesticides Associated to Atmospheric Aerosols: Hazard and Cancer Risk Assessments. *J. Braz. Chem. Soc.* **2020**, doi:10.21577/0103-5053.20200017.
59. Zhou, Y.; Guo, J.; Wang, Z.; Zhang, B.; Sun, Z.; Yun, X.; Zhang, J. Levels and Inhalation Health Risk of Neonicotinoid Insecticides in Fine Particulate Matter (PM2.5) in Urban and Rural Areas of China. *Environment International* **2020**, *142*, 105822, doi:10.1016/j.envint.2020.105822.
60. Ikenaka, Y.; Miyabara, Y.; Ichise, T.; Nakayama, S.; Nimako, C.; Ishizuka, M.; Tohyama, C. Exposures of Children to Neonicotinoids in Pine Wilt Disease Control Areas. *Environmental toxicology and chemistry* **2019**, *38*, 71–79.
61. Anh, H.Q.; Tomioka, K.; Tue, N.M.; Tuyen, L.H.; Chi, N.K.; Minh, T.B.; Viet, P.H.; Takahashi, S. A Preliminary Investigation of 942 Organic Micro-Pollutants in the Atmosphere in Waste Processing and Urban Areas, Northern Vietnam: Levels, Potential Sources, and Risk Assessment. *Ecotoxicology and Environmental Safety* **2019**, *167*, 354–364, doi:10.1016/j.ecoenv.2018.10.026.
62. Yu, S.Y.; Liu, W.J.; Xu, Y.S.; Zhao, Y.Z.; Cai, C.Y.; Liu, Y.; Wang, X.; Xiong, G.N.; Tao, S.; Liu, W.X. Organochlorine Pesticides in Ambient Air from the Littoral Cities of Northern China: Spatial Distribution, Seasonal Variation, Source Apportionment and Cancer Risk Assessment. *Science of The Total Environment* **2019**, *652*, 163–176, doi:10.1016/j.scitotenv.2018.10.230.
63. Pirard, C.; Remy, S.; Giusti, A.; Champon, L.; Charlier, C. Assessment of Children's Exposure to Currently Used Pesticides in Wallonia, Belgium. *Toxicology Letters* **2020**, *329*, 1–11, doi:10.1016/j.toxlet.2020.04.020.
64. Arcury, T.A.; Chen, H.; Quandt, S.A.; Talton, J.W.; Anderson, K.A.; Scott, R.P.; Jensen, A.; Laurienti, P.J. Pesticide Exposure among Latinx Children: Comparison of Children in Rural, Farmworker and Urban, Non-Farmworker Communities. *Science of The Total Environment* **2021**, *763*, 144233, doi:10.1016/j.scitotenv.2020.144233.
65. Yi, Y.J.; Joung, H.J.; Kum, J.Y.; Hwang, I.S.; Kim, M.S. Pesticide residues in vegetables and risk assessment for consumers in Korea during 2010–2014. *Food Additives & Contaminants: Part A* **2020**, 37(8), 1300-1313.

66. Nougadère, A.; Sirot, V.; Cravedi, J.-P.; Vasseur, P.; Feidt, C.; Fussell, R.J.; Hu, R.; Leblanc, J.-C.; Jean, J.; Rivière, G.; et al. Dietary Exposure to Pesticide Residues and Associated Health Risks in Infants and Young Children – Results of the French Infant Total Diet Study. *Environment International* **2020**, *137*, 105529, doi:10.1016/j.envint.2020.105529.
67. Bommuraj, V.; Chen, Y.; Klein, H.; Sperling, R.; Barel, S.; Shimshoni, J.A. Pesticide and Trace Element Residues in Honey and Beeswax Combs from Israel in Association with Human Risk Assessment and Honey Adulteration. *Food Chemistry* **2019**, *299*, 125123, doi:10.1016/j.foodchem.2019.125123.
68. Yi, Y.-J.; Joung, H.-J.; Kum, J.-Y.; Hwang, I.-S.; Kim, M.S. Pesticide Residues in Vegetables and Risk Assessment for Consumers in Korea during 2010–2014. *Food Additives & Contaminants: Part A* **2020**, *37*, 1300–1313.
69. Olisah, C.; Okoh, O.O.; Okoh, A.I. Distribution of Organochlorine Pesticides in Fresh Fish Carcasses from Selected Estuaries in Eastern Cape Province, South Africa, and the Associated Health Risk Assessment. *Marine Pollution Bulletin* **2019**, *149*, 110605, doi:10.1016/j.marpolbul.2019.110605.
70. Aydin, S.; Aydin, M.E.; Beduk, F.; Ulvi, A. Organohalogenated pollutants in raw and UHT cow's milk from Turkey: a risk assessment of dietary intake. *Environmental Science and Pollution Research*, **2019**, 26(13), 12788-12797.
71. El Agrebi, N.; Wilmart, O.; Urbain, B.; Danneels, E.L.; de Graaf, D.C.; Saegerman, C. Belgian Case Study on Flumethrin Residues in Beeswax: Possible Impact on Honeybee and Prediction of the Maximum Daily Intake for Consumers. *Science of The Total Environment* **2019**, *687*, 712–719, doi:10.1016/j.scitotenv.2019.05.493.
72. Elgueta, S.; Valenzuela, M.; Fuentes, M.; Meza, P.; Manzur, J.P.; Liu, S.; Zhao, G.; Correa, A. Pesticide Residues and Health Risk Assessment in Tomatoes and Lettuces from Farms of Metropolitan Region Chile. *Molecules* **2020**, *25*, 355, doi:10.3390/molecules25020355.
73. Marete, G.M.; Shikuku, V.O.; Lalah, J.O.; Mputhia, J.; Wekesa, V.W. Occurrence of pesticides residues in French beans, tomatoes, and kale in Kenya, and their human health risk indicators. *Environmental Monitoring and Assessment* **2020**, 192(11).
74. Sharma, K.K.; Tripathy, V.; Rao, C.S.; Bhushan, V.S.; Reddy, K.N.; Jyot, G.; Sahoo, S.K.; Singh, B.; Mandal, K.; Banerjee, H.; et al. Persistence, Dissipation, and Risk Assessment of a Combination Formulation of Trifloxystrobin and Tebuconazole Fungicides in/on Tomato. *Regulatory Toxicology and Pharmacology* **2019**, *108*, 104471, doi:10.1016/j.yrtph.2019.104471.
75. Lin, H.; Zhao, S.; Fan, X.; Ma, Y.; Wu, X.; Su, Y.; Hu, J. Residue Behavior and Dietary Risk Assessment of Chlorothalonil and Its Metabolite SDS-3701 in Water Spinach to Propose Maximum Residue Limit (MRL). *Regulatory Toxicology and Pharmacology* **2019**, *107*, 104416, doi:10.1016/j.yrtph.2019.104416.
76. Gong, J.; Zheng, K.; Yang, G.; Zhao, S.; Zhang, K.; Hu, D. Determination, Residue Analysis, Risk Assessment and Processing Factor of Pymetrozine and Its Metabolites in Chinese Kale under Field Conditions. *Food Additives & Contaminants: Part a* **2019**, *36*, 141–151.
77. Fan, X.; Zhao, S.; Hu, J. Dissipation Behavior and Dietary Risk Assessment of Lambda-Cyhalothrin, Thiamethoxam and Its Metabolite Clothianidin in Apple after Open Field Application. *Regulatory Toxicology and Pharmacology* **2019**, *101*, 135–141, doi:10.1016/j.yrtph.2018.11.003.
78. Dong, M.; Ma, L.; Zhan, X.; Chen, J.; Huang, L.; Wang, W.; Zhao, L. Dissipation Rates and Residue Levels of Diflubenzuron and Difenoconazole on Peaches and Dietary Risk Assessment. *Regulatory Toxicology and Pharmacology* **2019**, *108*, 104447, doi:10.1016/j.yrtph.2019.104447.
79. Buah-Kwofie, A.; Humphries, M.S.; Pillay, L. Dietary Exposure and Risk Assessment of Organochlorine Pesticide Residues in Rural Communities Living within Catchment Areas of ISimangaliso World Heritage Site, South Africa. *Environmental Science and Pollution Research* **2019**, *26*, 17774–17786.
80. Margenat, A.; Matamoros, V.; Díez, S.; Cañameras, N.; Comas, J.; Bayona, J.M. Occurrence and Human Health Implications of Chemical Contaminants in Vegetables Grown in Peri-Urban Agriculture. *Environment International* **2019**, *124*, 49–57, doi:10.1016/j.envint.2018.12.013.
81. Zhao, Z.; Sun, R.; Su, Y.; Hu, J.; Liu, X. Fate, Residues and Dietary Risk Assessment of the Fungicides Epoxiconazole and Pyraclostrobin in Wheat in Twelve Different Regions, China. *Ecotoxicology and Environmental Safety* **2021**, *207*, 111236, doi:10.1016/j.ecoenv.2020.111236.
82. Su, Y.; Wang, W.; Hu, J.; Liu, X. Dissipation Behavior, Residues Distribution and Dietary Risk Assessment of Tembotrione and Its Metabolite in Maize via QuEChERS Using HPLC-MS/MS Technique. *Ecotoxicology and Environmental Safety* **2020**, *191*, 110187, doi:10.1016/j.ecoenv.2020.110187.
83. Pang, K.; Hu, J. Simultaneous Analysis and Dietary Exposure Risk Assessment of Fomesafen, Clomazone, Clethodim and Its Two Metabolites in Soybean Ecosystem. *International journal of environmental research and public health* **2020**, *17*, 1951.
84. Souza, R.C.; Portella, R.B.; Almeida, P.V.N.B.; Pinto, C.O.; Gubert, P.; Silva, J.D.S. da; Nakamura, T.C.; Rego, E.L. do Human Milk Contamination by Nine Organochlorine Pesticide Residues (OCPs). *Journal of Environmental Science and Health, Part B* **2020**, *55*, 530–538, doi:10.1080/03601234.2020.1729630.
85. Sharma, N.; Chandel, R.; Sharma, I.; Sharma, P.; Gurung, B. Pesticides Contamination of Lactating Mothers' Milk in the North-Western Himalayan Region of India. *Journal of Environmental Biology* **2020**, *41*, 23–28.
86. Smadi, N.; Jammoul, A.; El Darra, N. Assessment of Antibiotic and Pesticides Residues in Breast Milk of Syrian Refugee Lactating Mothers. *Toxics* **2019**, *7*, 39, doi:10.3390/toxics7030039.
87. Müller, M.H.B.; Polder, A.; Brynildsrud, O.B.; Grønnestad, R.; Karimi, M.; Lie, E.; Manyilizu, W.B.; Mdegela, R.H.; Mokiti, F.; Murtadha, M.; et al. Prenatal Exposure to Persistent Organic Pollutants in Northern Tanzania and

Their Distribution between Breast Milk, Maternal Blood, Placenta and Cord Blood. *Environmental Research* **2019**, *170*, 433–442, doi:10.1016/j.envres.2018.12.026.
88. Hernández, A.F.; Lozano-Paniagua, D.; González-Alzaga, B.; Kavvalakis, M.P.; Tzatzarakis, M.N.; López-Flores, I.; Aguilar-Garduño, C.; Caparros-Gonzalez, R.A.; Tsatsakis, A.M.; Lacasaña, M. Biomonitoring of Common Organophosphate Metabolites in Hair and Urine of Children from an Agricultural Community. *Environment International* **2019**, *131*, 104997, doi:10.1016/j.envint.2019.104997.
89. Stajnko, A.; Snoj Tratnik, J.; Kosjek, T.; Mazej, D.; Jagodic, M.; Eržen, I.; Horvat, M. Seasonal Glyphosate and AMPA Levels in Urine of Children and Adolescents Living in Rural Regions of Northeastern Slovenia. *Environment International* **2020**, *143*, 105985, doi:10.1016/j.envint.2020.105985.
90. Lehmler, H.-J.; Simonsen, D.; Liu, B.; Bao, W. Environmental Exposure to Pyrethroid Pesticides in a Nationally Representative Sample of U.S. Adults and Children: The National Health and Nutrition Examination Survey 2007–2012. *Environmental Pollution* **2020**, *267*, 115489, doi:10.1016/j.envpol.2020.115489.
91. Jaacks, L.M.; Yadav, S.; Panuwet, P.; Kumar, S.; Rajacharya, G.H.; Johnson, C.; Rawal, I.; Mohan, D.; Mohan, V.; Tandon, N.; et al. Metabolite of the Pesticide DDT and Incident Type 2 Diabetes in Urban India. *Environment International* **2019**, *133*, 105089, doi:10.1016/j.envint.2019.105089.
92. Bassig, B.A.; Engel, L.S.; Langseth, H.; Grimsrud, T.K.; Cantor, K.P.; Vermeulen, R.; Purdue, M.P.; Barr, D.B.; Wong, J.Y.Y.; Blair, A.; et al. Pre-Diagnostic Serum Concentrations of Organochlorines and Risk of Acute Myeloid Leukemia: A Nested Case-Control Study in the Norwegian Janus Serum Bank Cohort. *Environment International* **2019**, *125*, 229–235, doi:10.1016/j.envint.2019.01.066.
93. Papadopoulou Eleni; Haug Line Småstuen; Sakhi Amrit Kaur; Andrusaityte Sandra; Basagaña Xavier; Brantsaeter Anne Lise; Casas Maribel; Fernández-Barrés Sílvia; Grazuleviciene Regina; Knutsen Helle Katrine; et al. Diet as a Source of Exposure to Environmental Contaminants for Pregnant Women and Children from Six European Countries. *Environmental Health Perspectives 127*, 107005, doi:10.1289/EHP5324.
94. Gracia-Lor, E.; Rousis, N.I.; Hernández, F.; Zuccato, E.; Castiglioni, S. Wastewater-Based Epidemiology as a Novel Biomonitoring Tool to Evaluate Human Exposure to Pollutants. **2018**.
95. Devault, D.A.; Karolak, S. Wastewater-Based Epidemiology Approach to Assess Population Exposure to Pesticides: A Review of a Pesticide Pharmacokinetic Dataset. *Environmental Science and Pollution Research* **2020**, *27*, 4695–4702.
96. Choi, P.M.; Tscharke, B.J.; Donner, E.; O'Brien, J.W.; Grant, S.C.; Kaserzon, S.L.; Mackie, R.; O'Malley, E.; Crosbie, N.D.; Thomas, K.V.; et al. Wastewater-Based Epidemiology Biomarkers: Past, Present and Future. *TrAC Trends in Analytical Chemistry* **2018**, *105*, 453–469, doi:10.1016/j.trac.2018.06.004.
97. Lorenzo, M.; Picó, Y. Wastewater-Based Epidemiology: Current Status and Future Prospects. *Current Opinion in Environmental Science & Health* **2019**, *9*, 77–84, doi:10.1016/j.coesh.2019.05.007.
98. Song, S.; Zhang, T.; Huang, Y.; Zhang, B.; Guo, Y.; He, Y.; Huang, X.; Bai, X.; Kannan, K. Urinary Metabolites of Neonicotinoid Insecticides: Levels and Recommendations for Future Biomonitoring Studies in China. *Environmental science & technology* **2020**, *54*, 8210–8220.
99. Fernández, S.F.; Pardo, O.; Adam-Cervera, I.; Montesinos, L.; Corpas-Burgos, F.; Roca, M.; Pastor, A.; Vento, M.; Cernada, M.; Yusà, V. Biomonitoring of Non-Persistent Pesticides in Urine from Lactating Mothers: Exposure and Risk Assessment. *Science of The Total Environment* **2020**, *699*, 134385, doi:10.1016/j.scitotenv.2019.134385.
100. Berman, T.; Barnett-Itzhaki, Z.; Göen, T.; Hamama, Z.; Axelrod, R.; Keinan-Boker, L.; Shimony, T.; Goldsmith, R. Organophosphate Pesticide Exposure in Children in Israel: Dietary Associations and Implications for Risk Assessment. *Environmental Research* **2020**, *182*, 108739, doi:10.1016/j.envres.2019.108739.
101. Hyland, C.; Kogut, K.; Gunier, R.B.; Castorina, R.; Curl, C.; Eskenazi, B.; Bradman, A. Organophosphate Pesticide Dose Estimation from Spot and 24-Hr Urine Samples Collected from Children in an Agricultural Community. *Environment International* **2021**, *146*, 106226, doi:10.1016/j.envint.2020.106226.
102. Faÿs, F.; Palazzi, P.; Hardy, E.M.; Schaeffer, C.; Phillipat, C.; Zeimet, E.; Vaillant, M.; Beausoleil, C.; Rousselle, C.; Slama, R.; et al. Is There an Optimal Sampling Time and Number of Samples for Assessing Exposure to Fast Elimination Endocrine Disruptors with Urinary Biomarkers? *Science of The Total Environment* **2020**, *747*, 141185, doi:10.1016/j.scitotenv.2020.141185.
103. F. Fernández, S.; Pardo, O.; Corpas-Burgos, F.; Yusà, V. Exposure and Cumulative Risk Assessment to Non-Persistent Pesticides in Spanish Children Using Biomonitoring. *Science of The Total Environment* **2020**, *746*, 140983, doi:10.1016/j.scitotenv.2020.140983.
104. Xu, Q.; Zhu, B.; Dong, X.; Li, S.; Song, X.; Xiao, X.; Zhang, C.; Lv, Y.; Zhang, X.; Li, Y. Pyrethroid Pesticide Exposure during Early Pregnancy and Birth Outcomes in Southwest China: A Birth Cohort Study. *J. Toxicol. Sci.* **2020**, *45*, 281–291, doi:10.2131/jts.45.281.
105. Zhang, T.; Song, S.; Bai, X.; He, Y.; Zhang, B.; Gui, M.; Kannan, K.; Lu, S.; Huang, Y.; Sun, H. A Nationwide Survey of Urinary Concentrations of Neonicotinoid Insecticides in China. *Environment International* **2019**, *132*, 105114, doi:10.1016/j.envint.2019.105114.
106. Tao, Y.; Dong, F.; Xu, J.; Phung, D.; Liu, Q.; Li, R.; Liu, X.; Wu, X.; He, M.; Zheng, Y. Characteristics of Neonicotinoid Imidacloprid in Urine Following Exposure of Humans to Orchards in China. *Environment International* **2019**, *132*, 105079, doi:10.1016/j.envint.2019.105079.
107. Li, A.J.; Martinez-Moral, M.-P.; Kannan, K. Temporal Variability in Urinary Pesticide Concentrations in Repeated-Spot and First-Morning-Void Samples and Its Association with Oxidative Stress in Healthy Individuals. *Environment International* **2019**, *130*, 104904, doi:10.1016/j.envint.2019.104904.

108. Muñoz-Quezada, M.T.; Lucero, B.; Bradman, A.; Steenland, K.; Zúñiga, L.; Calafat, A.M.; Ospina, M.; Iglesias, V.; Muñoz, M.P.; Buralli, R.J.; et al. An Educational Intervention on the Risk Perception of Pesticides Exposure and Organophosphate Metabolites Urinary Concentrations in Rural School Children in Maule Region, Chile. *Environmental Research* **2019**, *176*, 108554, doi:10.1016/j.envres.2019.108554.
109. Environmental Exposures in Young Adults with Declining Kidney Function in a Population at Risk of Mesoamerican Nephropathy \textbar Occupational & Environmental Medicine Available online: https://oem.bmj.com/content/76/12/920 (accessed on 6 March 2021).
110. English, K.; Li, Y.; Jagals, P.; Ware, R.S.; Wang, X.; He, C.; Mueller, J.F.; Sly, P.D. Development of a Questionnaire-Based Insecticide Exposure Assessment Method and Comparison with Urinary Insecticide Biomarkers in Young Australian Children. *Environmental Research* **2019**, *178*, 108613, doi:10.1016/j.envres.2019.108613.
111. Charisiadis, P.; Delplancke, T.; Makris, K. C. (2019). Cohort-friendly protocol for the determination of two urinary biomarkers of exposure to pyrethroids and neonicotinoids using gas chromatography-triple quadrupole mass spectrometry. *Analytical and bioanalytical chemistry* **2019**, 411(20), 5013-5021.
112. Kalloo, G.; Wellenius, G.A.; McCandless, L.; Calafat, A.M.; Sjodin, A.; Romano, M.E.; Karagas, M.R.; Chen, A.; Yolton, K.; Lanphear, B.P.; et al. Exposures to Chemical Mixtures during Pregnancy and Neonatal Outcomes: The HOME Study. *Environment International* **2020**, *134*, 105219, doi:10.1016/j.envint.2019.105219.
113. Han, X.; Zhang, F.; Meng, L.; Xu, Y.; Li, Y.; Li, A.; Turyk, M.E.; Yang, R.; Wang, P.; Zhang, J.; et al. Exposure to Organochlorine Pesticides and the Risk of Type 2 Diabetes in the Population of East China. *Ecotoxicology and Environmental Safety* **2020**, *190*, 110125, doi:10.1016/j.ecoenv.2019.110125.
114. Li, A.J.; Banjabi, A.A.; Takazawa, M.; Kumosani, T.A.; Yousef, J.M.; Kannan, K. Serum Concentrations of Pesticides Including Organophosphates, Pyrethroids and Neonicotinoids in a Population with Osteoarthritis in Saudi Arabia. *Science of The Total Environment* **2020**, *737*, 139706, doi:10.1016/j.scitotenv.2020.139706.
115. Savadatti, S.S.; Liu, M.; Caglayan, C.; Reuther, J.; Lewis-Michl, E.L.; Aldous, K.M.; Parsons, P.J.; Kannan, K.; Rej, R.; Wang, W.; et al. Biomonitoring of Populations in Western New York at Risk for Exposure to Great Lakes Contaminants. *Environmental Research* **2019**, *179*, 108690, doi:10.1016/j.envres.2019.108690.
116. Watkins, D.J.; Vélez-Vega, C.M.; Rosario, Z.; Cordero, J.F.; Alshawabkeh, A.N.; Meeker, J.D. Preliminary Assessment of Exposure to Persistent Organic Pollutants among Pregnant Women in Puerto Rico. *International Journal of Hygiene and Environmental Health* **2019**, *222*, 327–331, doi:10.1016/j.ijheh.2019.02.001.
